# The validity and reliability of the Mandarin Chinese version of the drug abuse screening test among adolescents in Taiwan

**DOI:** 10.1186/s13011-017-0109-2

**Published:** 2017-06-06

**Authors:** Jung-Yu Liao, Hsueh-Yun Chi, Jong-Long Guo, Chiu-Mieh Huang, Shu-Fang Shih

**Affiliations:** 1 Department of Health Promotion and Health Education, School of Education, National Taiwan Normal University, No. 162, Section 1, Heping East Road, Taipei, ROC Taiwan; 2 Health Healing and Health Marketing, Kainan University, No.1, Kainan Road, Luzhu District, Taoyuan, R.O.C. Taiwan; 3 Institute of Clinical Nursing, National Yang Ming University, No.155, Sec.2, Linong Street, Taipei, R.O.C. Taiwan

**Keywords:** Adolescents, Drug abuse screening test, Reliability, Validity, Drug use

## Abstract

**Background:**

This study aimed to investigate the validity and reliability of the Mandarin Chinese version of the Drug Abuse Screening Test (DAST-20) among adolescents, as well as examining the test’s predictability with regard to the actual level of drug abuse.

**Method:**

A total of 100 adolescent participants were recruited with their consent, in which 49 were current drug users and 51 were non-users. Based on the frequency of their drug use, participants who had used drugs at least once every week during the past year were classified as regular users (34 participants); participants who had not reached the regular use frequency of once every week during the past year were classified as occasional users (15 participants). All of the participants were required to answer a sociodemographic questionnaire, and undergo a DAST-20 (Mandarin Chinese version).

**Results:**

The DAST-20, which has a high reliability with a Cronbach’s alpha of 0.88, and a construct validity accounting for 61.87% of the variance. The area under the receiver operating characteristics (ROC) curve was 0.96 between the users and nonusers, and 0.93 between the occasional users and non-occasional users. A cut-off of three points could identify 93.5% of the drug users and 88.9% of nonusers, whereas a cut-off of six points could identify 85.3% of the regular users and 92.4% of non-regular users.

**Conclusions:**

The DAST-20 was the strongest predictor of adolescents who were regular users, occasional users, and non-users after controlling for other potential covariates. The Mandarin Chinese version of the DAST is simple to use and has a satisfactory validity and reliability. It is an effective screening tool for drug users among adolescents.

## Background

Using illegal drugs not only causes severe health problems [1, 2], but is also highly correlated with social problems such as traffic accidents, violence, and theft [3]. A predilection for illegal drug use often occurs in adolescence [4]. Because illegal drugs are addictive, their usage during adolescence will therefore continue into adulthood [5]. Additionally, the prevalence of illegal drug use among adolescents has substantially increased in recent years [6]. Therefore, illegal drug use among adolescents has now become a crucial topic for discussion.

The prevalence of illegal drug use within one month among adolescents in Taiwan is slowly increasing [7]. Currently, approximately 1.06% of adolescents are known to have used illegal drugs, among whom high school students have exhibited the most sever condition (prevalence = 1.97%), followed by college students (prevalence = 1.09%). Students in the continuing education programs in vocational schools had a comparatively higher prevalence of 4.37% [8]. So far, Taiwan’s government has used the urine screening tests as indicators of illegal drug use to identify drug users, however, an effective screening scale for distinguishing between drug users and nonusers is still sadly lacking.

A study has indicated that a combination of early detections and short-term interventions is an effective method for changing adolescent’s substance use [9]. Various drug use screening scales have been developed for the early identifications of drug use in foreign countries. So far, some screening scales have been developed such as Michigan Alcoholism Screening Test (MAST) [10], Alcohol Use Disorders Identification Test (AUDIT) [11], CAGE-AID (Adapted to Include Drugs) [12], Composite International Diagnostic Interview (CICD) [13], and the alcohol, smoking and substance involvement screening test (ASSIST) [14]. Among these screening scales, MAST and AUDIT were related to alcohol use, ASSIST included more than one substance uses, and CAGE-AID contains only four questions regarding alcohol and other drugs. On the other hand, Drug Abuse Screening Test (DAST), and the Cannabis Abuse Screening Test (CAST) mainly focus on illegal drugs [15].

The DAST is an internationally well-recognized scale for the identifications of drug use [16], modeled from the MAST. The test contains three versions (DAST-28, DAST-20, and DAST-10), that have excellent validity and reliability, and are widely used for both professionals and the general public [17]. There are several studies using DAST to identify the drug use among adolescents. The positive rates of the common drugs of abuse detected in samples collected from participants in a disco were as follows: MDMA, 75.7%; ketamine, 47.0%; MA, 41.6%; opiates, 0%. Marijuana and cocaine were detected at much lower rates (3.4 and 4.7%, respectively). Ketamine and one of the amphetamines were detected together in 42.9% of the samples. The positive rates in samples collected from police detainees suspected of drug abuse in the general public were as follows: MA, 76.0%; opiates, 37.0%; MDMA, 6.0%; ketamine, 2.0% [18].

A total of 2,126 adolescents aged from 12 to 18 years recruited from Taipei street sites completed a self-administered anonymous questionnaire. The lifetime prevalence of illicit drug use for adolescents with truancy was 15.0–17.9% (12.1–14.5% for ecstasy, 4.6–7.3% for ketamine, and 3.5–8.8% for marijuana), and the corresponding estimate was 3.1–3.4% for youths without truancy [19].

In terms of lifetime prevalence and incidence, ecstasy and ketamine by and large appeared as the first and second commonly used illegal drugs, respectively, among middle (grades 7 to 9) and high school students (grades 10 to 12), during the 3-year survey period; however, this order was reversed in the middle school-aged students starting in 2006. Having sexual experience, tobacco use, and betel nut use were factors consistently associated with the onset of ecstasy use across years. The majority of ecstasy users had been involved in polydrug use, such as the use of ketamine (41.4%–53.5%), marijuana (12.7%–18.7%), and methamphetamine (4.2%–9.5%) [20].

Since DAST is more general, rather than focusing on one illegal drug such as CAST, it is therefore more suitable in Taiwan as a screening tool. We obtained authorization from the original author and developed a Mandarin Chinese version of the DAST-20, tested its validity and reliability among adolescents, and examined the predictability regarding the abuse level of various drugs. By using this effective and simple screening scale in Taiwan, illegal drug usage and levels of use among adolescents can be accurately ascertained in a timely manner, and appropriate intervention programs can be implemented accordingly for reducing illegal drug usage rates.

## Methods

### Participants

Both drug users and nonusers were included as the participants. Furthermore, because illegal drug usage is a sensitive topic and users are rarely willing to admit to either being one and/or talk about it, all the participants in this study were recruited from an occupational school, with the participates in a drug abuse counseling program where only users were invited to participate in their drug abuse counseling program. After the research plan was approved by the school administration, the program counselors provided the students’ names who had used illegal drugs (those classified in Categories 1–4, specified in the Narcotics Hazard Prevention Act [21]; e.g., club drugs such as ketamine, ecstasy, and amphetamine) within the past 30 days, and produced positive urine screening results. In addition, the participants had no difficulties in answering the questionnaire, had no visual or hearing impairment, and were able to speak and understand Mandarin and/or Taiwanese. The nonuser participants were recruited from different classes within the same school. The characteristics of the non-users were similar to the group of users in terms of educational level of the primary guardians and family structure, except for gender and age. This group exhibited no symptoms of drug use, self-reported that they were not using drugs, and were confirmed by teachers as exhibiting no symptoms or problems related to drug use. Consent forms were obtained from all of these participants. Finally, a total of 100 students consented to participate in this study, among whom 49 were users and 51 were nonusers of illegal drug.

### Measures

The Mandarin Chinese version of the DAST-20 was adopted as the research tool in this study. The symptoms of substance dependence, such as withdrawal, as well as social and emotional problems related to substance use, were included in the DAST scale. Two experts on addiction were invited to translate the DAST scale into Chinese after obtaining the author’s permission. We followed the six steps suggested by the World Health Organization (WHO) to develop our Mandarin Chinese version of the English instrument on the management of substance abuse. The six steps were forward translation, review by an expert panel, back-translation, pretesting and cognitive interviewing, creation of the final version, and documentation [22]. In addition, this version of the DAST was pilot tested by three high school students, one was a nonuser and the other two were users, and the wording was modified to fit in with the Taiwanese adolescent sociocultural context.

The 100 participants were invited to complete this version of the DAST-20, and the total score for the Mandarin Chinese version of the DAST-20 ranged from 0 to 20; a score of 1 point was given for each question answered “yes,” except for Questions 4 and 5, for which a “no” received 1 point.

The participants also provided their background information including age, gender, education level of their primary guardian (junior high or below and high school and above), family structure (living with their parents or others), communication style (discussion-based, authoritarian, and permissive styles), and drug use frequency (for drug users only). The data regarding drug use frequency were obtained based on the participants’ self-reported times of drug use within the past year. Participants who had used drugs at least once every month, but had not reached a frequency of once every week during the past year, were categorized as occasional users, whereas participants who had used drugs at least once every week during the past year were categorized as regular users [23].

### Statistical analysis

In order to test the validity and reliability of the DAST-20 to identify the illegal drug users, we recruited drug users and non-drug users in our research. We used SPSS software for Windows, version 17.0 (SPSS Inc., Chicago, IL, USA) to conduct a *t* test and/or a chi-square test for comparing the sociodemographic differences between the drug user and nonuser groups. The Cronbach’s alpha was an indicator of the internal consistency, and the principal component analysis (PCA) was used to examine the factor structure of this DAST-20 version. The method for rotation was VARIMAX. Furthermore, the receiver operating characteristic (ROC) analysis was generated and the area under the ROC curve was used for determining the distinguishing ability of the scale. The sensitivities, specificities, positive predictive values, and negative predictive values, at various cut-off points were individually presented. The optimal cut-off point was determined using Kappa and χ^2^ for the power of discrimination [24]. In order to examine whether the scale could predict the drugs or substance use, we used a hierarchical logistic regression. In the first step, we included the background information, and the scale in the second step.

## Results

### Sociodemographic characteristics of participants

The participants were divided into drug users and nonusers. A comparison of their sociodemographic data is presented in Table 1, with age (t = 5.39, *p <* 0.001), gender (χ^2^ = 8.58, *p =* 0.003), communication style (χ^2^ = 7.20, *p =* 0.007), and the DAST-20 score (t = 11.85, *p <* 0.001), were statistically significant between these two groups. The variables of the education level of the primary guardian (χ^2^ = 1.20, *p =* 0.274), and whether the participants lived with their parents (χ^2^ = 0.00, *p =* 0.968), was not statistically different between these two groups.

**Table 1:** Sociodemographic characteristics of the participants

Variable	Drug user		Nonuser		t/ χ^2^	*p*
	n	mean (±SD)/ %	n	mean (±SD)/ %		
Age	47	17.40 (±1.50)	51	16.12 (±0.68)	5.39	<0.001
Gender					8.58	0.003
Male	43	89.58	33	64.71		
Female	5	10.42	18	35.29		
Education level of the primary guardian					1.20	0.274
Junior high school or below	15	31.25	11	21.57		
High school and above	33	68.75	40	78.43		
Family structure					0.00	0.968
Living with parents	33	70.21	36	70.59		
Other^a^	14	29.79	15	29.41		
Communication style					7.20	0.007
Discussion-based	28	59.57	42	84.00		
Other^b^	19	40.43	8	16.00		
Regular user					--	--
Yes	34	69.39	0	0.00		
No	15	30.61	51	100.00		
DAST-20 score	49	7.22 (±3.67)	51	0.78 (±1.03)	11.85	<0.001

Note

Continuous variables (age and DAST-20 score) were tested using t test; df of age = 54; df of DAST-20 score = 54

Categorical variables (gender, educational level of the primary guardian, family structure, communication style and regular user) were tested using chi-test; df = 1

^a^Including single-parent, grandparent families, and so on ^b^ Including authoritarian and permissive styles

### Reliability and validity of the Mandarin Chinese version of the DAST-20

The Mandarin Chinese version of the DAST-20 has a high internal consistency, with a Cronbach’s alpha of 0.88. Bartlett’s test of sphericity (χ^2^ = 1152.66, *p <* 0.001, df = 190), and the Kaisar–Meyer–Olkin value (0.73), supported the factorability of the correlation matrix. The results of the PCA revealed four components, accounting for 61.87% of the variance. The initial (unrotation) eigenvalue of the components was 6.73, 2.67, 1.66, and 1.32 respectively. The item-loading for factor 1 was 0.578-0.823, 0.550-0.854 for factor 2, 0.450-0.773 for factor 3, 0.808-0.854 for factor 4. It has been shown that the first factor is the most important one among al. In addition, the values of the Cronbach’s alpha of four factors were 0.89, 0.83, 0.72, and 0.67, respectively,showing that the internal validity is more appropriate.

### Validity of the Mandarin Chinese version of the DAST-20

**Fig. 1:**
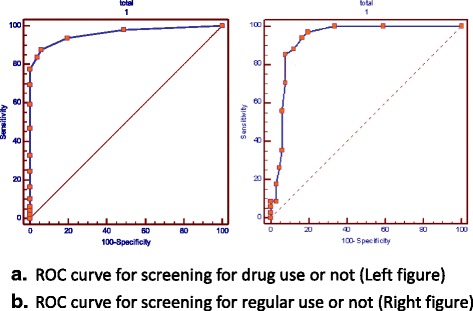
ROC curves for the screening scale: **a** drug use or not (*Left*); **b** regular drug use or not (*Right*)

The area under the ROC curve (Fig. 1) was 0.96 between the users and nonusers, and 0.93 between the regular users and non-regular users. The sensitivity and specificity of the cut-off points are shown in Table 2. A cut-off of three points had the highestχ^2^ and kappa values, where 93.48% of users and 88.89% of nonusers were correctly identified. Furthermore, the highestχ^2^ and kappa values were obtained when the cut-off point was six points, where 85.29% of regular users, and 92.42% of non-regular users were correctly identified.

**Table 2:** Scale scores for screening various levels of drug dependence

Score	Sensitivity %	Specificity %	PPV %	NPV %	kappa	χ^2^	*p*
Drug user vs Nonuser							
≧2	93.88	80.39	82.14	93.18	0.74	55.94	<0.001
≧3	87.76	94.12	93.48	88.89	0.82	67.44	<0.001
≧4	83.67	96.08	95.35	85.96	0.80	64.85	<0.001
Regular user vs Non-regular user							
≧5	88.24	87.88	78.95	93.55	0.70	48.91	<0.001
≧6	85.29	92.42	85.29	92.42	0.73	53.67	<0.001
≧7	70.59	92.42	82.75	85.92	0.65	43.27	<0.001

Note

*PPV* Positive Predictive Value, *NPV* Negative Predictive Value; All df = 1

Our study tested the associations between the DAST and the urine tests. The results showed that the correlation was 0.831 (*P <* 0.001). It has been shown that the subject measurement such as the DAST and the objective measurement such as the urine test, had a high correlation (>0.80).

### Predictors of drug use

Table 3 shows the results of the hierarchical logistic regression for predicting drug use. The DAST-20 was entered at step 2 after the other predictors and significantly predicted drug use and abuse among adolescents (drug user: Wald Chi-square = 27.68, odds ratio (OR) = 146.85, *p <* 0.001, df = 1; regular user: Wald Chi-square = 20.88, OR = 50.01, *p <* 0.001, df = 1). Age also significantly predicted adolescents who were drug users or nonusers (Wald Chi-square = 6.88, OR = 3.20, *p =* 0.009, df = 1). The DAST-20 displayed the strongest predictive power after controlling for other predictors.

**Table 3:** Hierarchical logistic regression for predicting drug use with the DAST-20 after controlling for other potential predictors

Variable	Wald	p	OR	95% CI
Drug user vs nonusers				
Age	6.88	0.009	3.20	1.34-7.62
Gender^a^	0.37	0.543	1.89	0.24-14.59
Communication style^b^	1.67	0.197	0.26	0.03-2.02
DAST-20 score ≧ 3	27.68	<0.001	146.85	22.89-941.98
Regular user vs others				
Age	0.65	0.421	0.78	0.42-1.44
Gender^a^	0.85	0.357	2.38	0.38-15.11
Communication style^b^	2.14	0.143	0.33	0.08-1.45
DAST-20 score ≧ 6	20.88	<0.001	50.01	9.34-267.75

Note

^a^Reference group: female; ^b^ Reference group: authoritarian and permissive communication styles

^c^drug user: Nagelkerke R^2^ = 0.831, regular user: Nagelkerke R^2^ = 0.653; All df = 1

## Discussion

The DAST is mainly used for screening illegal drug addiction or dependence. It has a satisfactory validity and reliability among adults [25, 26] and adolescents [27]. However, empirical studies on the validity and reliability of the Mandarin Chinese version of the DAST for adolescents are still lacking. This study has filled this gap and demonstrated that the DAST scale also had a satisfactory validity and reliability for screening illegal drug use among high school students.

According to previous studies, a score of six and above on the DAST-20 is indicative of illegal drug addiction. However, [28], reported a different finding. They considered that the DAST may have had several limitations in predicting a diagnosis of drug addiction for patients whose scores fall between three and six points. The results of this study indicated that the optimal cut-off points were influenced by the participants’ levels of drug abuse. According to the findings of Cocco and Carey [25], current abusers, former abusers, and thosewho have never met the criteria for a drug abuse, or drug dependence diagnosis, had clearly different DAST scores. The results of this study indicated that the cut-off point of six is a suitable indicator for drug abuse, and that of three is a suitable indicator for adolescent drug use because the 6-point cut-off correctly identified 85.3% of regular users, 92.4% of non-regular users, and the 3-point cut-off correctly identified 93.5% of drug users and 88.9% of nonusers.

The clinical importance of screening students possibly engaged in drug use resides in the subsequent interventions for preventing the development of chronic drug abuse or dependence, thereof. Prevention programs for substance addiction should be formulated based on the conditions of the target groups [29]. In other words, the selective prevention and the indicated prevention strategies, instead of a universal prevention program, should be adopted for those students exposed to either high risk, or those who have previously used drugs [30]. The DAST has been used for determining possible drug abuse among college students so as to provide a service for students who are considered to be at a high risk for intended drug abuse [31]. According to the results of this study, regular users who scored six or above on the DAST, and used drugs at least once every week, were exhibiting early signs of dependence. Students who scored 3–5 were approaching the occasional user category. Suitable interventions should be adopted for assisting students in ceasing illegal drug use because one dollar invested in preventing drug abuse generates a profit of 10 dollars [29].

In addition, research has indicated that sociodemographic characteristics such as increasing age, male gender, low education level of the primary guardian, single-parent family, and communication style, are highly correlated with illegal drug use [32]. Although these characteristics are important risk factors that have been identified in previous studies, conducting clinical research on them is difficult because it requires considerable time and effort, and students may object to directly answering related questions. Therefore, the practical applications of these characteristics still require further investigation. This study adopted a hierarchical logistic regression to compare the differences in the predictions of drug use by using various sociodemographic variables and the DAST-20 scale. The results indicated that the DAST-20 was the strongest predictor of drug use, and regular use after controlling for other variables and predictors. Simply stated, even if the risk factors based on the sociodemographic variables were not ascertained, the DAST used on its own for screening adolescents at high risk could still effectively discriminate against the occasional drug users from the regular users.

The DAST was suggested as an unidimensional scale in guide for using the drug abuse screening test [33], however, the factors found using the DAST-20 were around 1–6 [17] [34], however, our research found that there were four factors. In fact, some other confounding factors may increase the bias, and the sample size was one. Since the drug users among adolescents are a hard-to-touch population, and our study had recruited at least 100 cases based on the suggestions. [35]. Any possibility of a good recovery of the population factors probably requires very large samples, well over 500, but only two papers had used such as large sample so far [34, 36], and both approved that the DAST is a single-factor scale. Although our study only selected one school, however, this school took in all the students that had dropped out from other schools in neighboring counties. Therefore, our study included most of the drug users.

Our study had some limitations. First, our study only selected one school that participated in the drug abuse counseling program. The reason for this was that the occupational school contained the largest number of drug users had been repelled (quitted) by the other vocational schools because of using illicit drugs. Second, our study did not control for other behavioral factors such as smoking, alcohol drinking or betel quid chewing due to the lack of these information. However, since our sample size was relatively small, it was not recommended that we included too many variables in our analysis.

Currently, student drug testing (SDT) has been adopted in Taiwan as a school procedure to confirm if a student uses illicit drugs. In other words, SDT was implemented in response to the suspicion of use among high-risk students. SDT is divided into urine screening tests, and rapid screening reagents that provide solid confirmation of drug usage. However, SDT requires expenditure, which have been estimated based on the current market prices as follows, a single urine screening test costs NT$1,000, a high number of screening tests costs NT$400–500 each, and the prices of the rapid screening reagents vary for different types of drugs. Regarding the most frequently used illegal drugs by adolescents in Taiwan, a ketamine test costs approximately NT$60–65, an amphetamine or ecstasy test costs approximately NT$50, and a three-in-one (ketamine, amphetamine, and ecstasy) rapid screening reagent costs approximately NT$150–160. Generally speaking, students tend to resist the SDT. When compared with the SDT, the DAST is an indicator for drug use that can reduce costs and determine the level of drug use. Additionally, as a simple-to-use scale that does not require professional instructions, the DAST is relatively more acceptable among adolescents and, therefore, is suitable for large-scale tests.

In the future, the DAST-20 can be applied to high school and college students. Additionally, the Mandarin Chinese version of the DAST can be used as an indicator for student drug abuse during any relevant counseling. Students who score six or above have been identified as regular drug users, with a considerably higher probability of developing drug dependence in the future. Consequently, suitable prevention programs should be implemented for increasing the effectiveness of drug rehabilitation, reducing costs, and fulfilling the public health principles of early detection and treatment.

## Conclusions

This is the first study that aimed to investigate the validity and reliability of the Mandarin Chinese version of the Drug Abuse Screening Test (DAST-20) among adolescents, and to examine the test’s predictability with regard to the actual level of drug abuse. Our study has demonstrated that the DAST-20, which has a high reliability with a Cronbach’s alpha of 0.88 and a satisfactory construct validity. A cut-off of three points could identify 93.5% of drug users and 88.9% of nonusers (*p* < 0.001), whereas a cut-off of six points could identify 85.3% of regular users and 92.4% of non-regular users (*p* < 0.001). In addition, the DAST-20 was the strongest predictor of adolescents who were regular users, occasional users, and non-users of drugs after controlling for other potential covariates. Therefore, we suggested that the Mandarin Chinese version of the DAST can be used as an effective screening tool for schools to identify the drug users among the adolescents, and thereby take actions to help the drug users as early as possible.

## Abbreviations

MDMA: 3,4-Methylenedioxymethamphetamine, also known as ecstasy, is a psychoactive drug used primarily as a recreational drug
MA: Methamphetamine (contracted from N-methylamphetamine) is a strong central nervous system stimulant that is mainly used as a recreational drug
